# Rhinovirus Exacerbates House-Dust-Mite Induced Lung Disease in Adult Mice

**DOI:** 10.1371/journal.pone.0092163

**Published:** 2014-03-14

**Authors:** Jennifer A. Phan, Anthony Kicic, Luke J. Berry, Lynette B. Fernandes, Graeme R. Zosky, Peter D. Sly, Alexander N. Larcombe

**Affiliations:** 1 Division of Clinical Sciences, Telethon Institute for Child Health Research, The University of Western Australia, Subiaco, Western Australia, Australia; 2 Pharmacology, Pharmacy and Anaesthesiology Unit, School of Medicine and Pharmacology, The University of Western Australia, Crawley, Western Australia, Australia; 3 School of Paediatrics and Child Health, The University of Western Australia, Subiaco, Western Australia, Australia; 4 Department of Respiratory Medicine, Princess Margaret Hospital for Children, Perth, Western Australia, Australia; 5 Centre for Cell Therapy and Regenerative Medicine, School of Medicine and Pharmacology, The University of Western Australia, Crawley, Western Australia, Australia; 6 Queensland Children's Medical Research Institute, The University of Queensland, Brisbane, Queensland, Australia; University Hospital Freiburg, Germany

## Abstract

Human rhinovirus is a key viral trigger for asthma exacerbations. To date, murine studies investigating rhinovirus-induced exacerbation of allergic airways disease have employed systemic sensitisation/intranasal challenge with ovalbumin. In this study, we combined human-rhinovirus infection with a clinically relevant mouse model of aero-allergen exposure using house-dust-mite in an attempt to more accurately understand the links between human-rhinovirus infection and exacerbations of asthma. Adult BALB/c mice were intranasally exposed to low-dose house-dust-mite (or vehicle) daily for 10 days. On day 9, mice were inoculated with human-rhinovirus-1B (or UV-inactivated human-rhinovirus-1B). Forty-eight hours after inoculation, we assessed bronchoalveolar cellular inflammation, levels of relevant cytokines/serum antibodies, lung function and responsiveness/sensitivity to methacholine. House-dust-mite exposure did not result in a classical TH_2_-driven response, but was more representative of noneosinophilic asthma. However, there were significant effects of house-dust-mite exposure on most of the parameters measured including increased cellular inflammation (primarily macrophages and neutrophils), increased total IgE and house-dust-mite-specific IgG_1_ and increased responsiveness/sensitivity to methacholine. There were limited effects of human-rhinovirus-1B infection alone, and the combination of the two insults resulted in additive increases in neutrophil levels and lung parenchymal responses to methacholine (tissue elastance). We conclude that acute rhinovirus infection exacerbates house-dust-mite-induced lung disease in adult mice. The similarity of our results using the naturally occurring allergen house-dust-mite, to previous studies using ovalbumin, suggests that the exacerbation of allergic airways disease by rhinovirus infection could act via multiple or conserved mechanisms.

## Introduction

It has been known for over forty years that respiratory tract viral infections are a key trigger of exacerbations of respiratory conditions such as bronchitis [1], [2] and asthma [3]. With the advent of more specific diagnostic technologies such as RT-PCR, it became evident that a significant proportion of asthma exacerbations and hospital admissions for asthma were associated with a human rhinovirus (HRV) infection [4]. Further, these technologies confirmed that HRV is not just an infection of the upper respiratory tract, but rather that it is able to infect and replicate in the lower airways [5]. The association between HRV infection and asthma exacerbation has been observed in both children [6], [7], [8] and adults [9], [10].

Many mechanisms of HRV-induced exacerbation of asthma have been suggested, including altered pulmonary inflammation/cytokine profiles [11], increased susceptibility of asthmatic patients to HRV infection [12] and HRV-induced damage to the airway epithelium [13]. Indeed, controlled infection studies in humans have shown increased airway inflammation, and more severe coryzal symptoms, such as wheeze, in HRV-infected asthmatics [5], [14]. However, further investigation into these potential mechanisms has been slow due to the lack of suitable *in vivo* models which combine HRV infection and allergic airways disease. Previous *in vivo* studies have infected mice with a minor group virus, most notably HRV-1B, and systemically sensitised/intranasally challenged them with ovalbumin [15], [16], [17]. HRV-1B is closely related to HRV-16 [18], the serotype most often used in human infection studies [19]. BALB/c mice infected with HRV-1B develop rapid neutrophilic inflammation as well as peribronchial/perivascular cellular infiltration of macrophages and lymphocytes [15], [16]. Mice previously sensitised and then challenged with ovalbumin and infected with HRV-1B show increases in cellular inflammation, lung expression of cytokines including eotaxin-1, IL-4, IL-13 and IFN-γ, mucus secretion and respiratory system resistance (R_rs_) compared with controls [15], [16]. In many of these studies, neutrophilic inflammation of the lower airways was demonstrated to be a feature of asthma exacerbations [15], [16], [17], [20], [21], [22], [23], [24]. Variations on the murine ovalbumin model of allergic airways disease have been used for many years, despite some recent concerns about their applicability to the human condition [25], [26]. In particular, mice systemically sensitized to ovalbumin in conjunction with aluminium hydroxide and then challenged with inhaled ovalbumin do not exhibit epithelial damage and remodelling as seen in asthma sufferers. To address this, we exposed mice to house dust mite (HDM; *Dermatophagoides pteronyssinus*) prior to HRV-1B infection. HDM exposure has some advantages over traditional ovalbumin models in that it is a naturally occurring allergen which causes atopic sensitization (via the nasal mucosa), allergic airways disease, and damage to the airway epithelium in humans [27], [28], [29]. High protein content HDM also induces an asthmatic phenotype in mice, including elevated IgE and TH_2_ cytokines, eosinophila, increased airway smooth muscle, collagen deposition and increased R_rs_ [27], [30] without the need for chemical adjuvants.

In this study, we aimed to investigate the effects of HRV-1B infection on HDM-induced airways disease. This was achieved by exposing adult female BALB/c mice to low doses of HDM via the nasal mucosa prior to infection with HRV-1B. Forty-eight hours after infection, we assessed bronchoalveolar lavage inflammation and lung function. We hypothesised that mice sensitised to HDM and then infected with HRV-1B would exhibit greater impairments in lung function, and greater pulmonary inflammation compared with mice subjected to either insult alone. Our study is differentiated from previous works in this field by our choice of allergic airways disease model, in addition to the use of the constant phase model to partition respiratory system impedance into compartments representing the airways and lung parenchyma [31].

## Materials and Methods

### Ethics statement

This study was carried out in strict accordance with the recommendations of the Australian Code of Practice for the Care and Use of Animals for Scientific Purposes (7th Edition). The protocol was approved by The Telethon Institute for Child Health Research Animal Ethics Committee (approvals #216 and #220). All surgeries were performed under general anaesthesia and all efforts were made to minimize suffering.

### Animals

Adult female (7-weeks) BALB/c mice were obtained from Animal Resources Centre (Murdoch, WA, Australia) and housed at the Telethon Institute for Child Health Research (TICHR) in specific pathogen-free environments. Mice were maintained on a 12:12 hour light:dark cycle and supplied with an allergen free diet (Specialty Feeds, Glen Forrest, WA, Australia) and acidified water *ad libitum*.

### House dust mite sensitisation

Mice were lightly anaesthetised with methoxylflurane (Medical Development International Ltd, Springvale, Victoria, Australia) and intranasally inoculated with 25 μg of *Dermatophagoides pteronyssinus* protein (HDM: 17.35% w/w protein, 12.47 EU/mg; Greer Laboratories, Lenoir, NC, USA) dissolved in 50 μL of saline or saline alone (vehicle) by pipetting drops onto the nostrils until aspirated. This is the equivalent of approximately 144 μg of whole-crushed HDM. Mice received inoculations for ten consecutive days as previously described [27].

### Virus and infection

A laboratory strain of rhinovirus, HRV-1B was kindly provided by Prof. Peter Wark (Hunter Medical Research Institute, Newcastle, NSW. Australia). We used HRV-1B (a minor group HRV) as it binds to members of the low density lipoprotein (LDL) receptor family in mice [15], [32]. Since mice lack the intercellular adhesion molecule 1 (ICAM-1) receptor utilised by the majority of HRV serotypes only minor-group HRV serotypes are able to cause infection in this species [33].

HRV-1B was propagated on HeLa cells as described previously [34], [35]. After 24 hours of incubation at 35°C, 5×10^8^ infected cells were detached using a cell scraper and resuspended in PBS to a total volume of 15 mL. Cells were then lysed with the addition of 10% (v/v) nonyl phenoxypolyethoxylethanol (NP-40), and cell debris pelleted by centrifugation at 10,000 rpm at 4°C for 10 minutes. Supernatant fluid was supplemented with the addition of 200 μg RNase A and incubated at 35°C for 20 minutes to disrupt ribosomes. HRV-1B was then pelleted utilizing 30% sucrose cushion as described previously [35]. The viral pellet was resuspended in PBSa [phosphate buffered saline containing 0.01% Bovine Serum Albumin (BSA)], layered on two linear 5 mL gradients (7.5 to 45% sucrose in PBSa) and centrifuged for 2 hours at 16°C at 40,000 rpm. The purified viral band was then removed from the gradient using a Pasteur pipette and stored at −80°C. Virus concentration was measured using Nanodrop spectrophotometer OD260 measurement as previously described [36]. Virus infectivity was determined using MRC5 cells and calculated using the Spearman-Karber estimation of TCID_50_ [37].

Under light methoxyflurane anaesthesia mice were intranasally inoculated with 1×10^8^ TCID_50_ HRV-1B in 50 μL Dulbecco's Modified Eagle Medium (DMEM; Sigma-Aldrich Pty Ltd, Sydney, Australia) or inactivated virus (control) in DMEM. This was done 48 hours prior to study. Virus was inactivated by exposing it to UV-light for 3 hours and heating it to 57°C for 2 hours.

### Treatment groups

Mice were randomly assigned to one of four treatment groups: exposed to house dust mite and infected with human rhinovirus-1B (HDM-HRV *n* = 10); exposed to house dust mite and inactivated human rhinovirus-1B (HDM-iHRV *n* = 9); not exposed to house dust mite and infected with human rhinovirus1B (Sal-HRV *n* = 9) or not exposed to house dust mite or live human rhinovirus-1B (Sal-iHRV *n* = 8).

### Confirmation of infection

The lungs of all mice were inflation fixed with formalin and embedded in paraffin for immunohistochemical staining. Sections 5 μm thick were taken at 500 μm intervals from a random starting point, and were deparaffinised and rehydrated in xylene and graded ethanol. Antigen retrieval was performed using 10 mM citrate buffer (pH 7.0) in a pressure cooker at 120°C. Sections were permeabilised in TBS-Tween for 30 minutes before blocking of endogenous peroxidise activity with 3% H_2_O_2_ for 10 minutes. After rinsing in TBS-Tween, slides were blocked in 10% (v/v) normal goat serum for 30 minutes. HRV-1B antisera (American Type Culture Collection, Manassas, VA, USA) was diluted to 1:500 in blocking buffer and added to the slides overnight at 4°C, followed by incubation with biotinylated goat anti-rabbit IgGs (1:500) and avidin-peroxidase complex (Vector Laboratories, Peterborough, UK). Finally sections were developed with diaminobenzidine substrate and counterstained with 10% haematoxylin. Images were visualized at 20× magnification under light microscopy using an Olympus BX43 System Microscope (Olympus Australia Pty. Ltd., Mount Waverely, Australia).

### Lung function and responsiveness to methacholine

All *in vivo* studies were conducted 48 hours after HRV-1B inoculation, which coincided with 24 hours after the last HDM exposure (the “day-of-study”). This timing was based on preliminary kinetics studies which showed that the peak of HRV-1B induced pulmonary inflammation occurs 48 hours post infection in adult female BALB/c mice (Figure S1). Mice were surgically prepared, and lung function/responsiveness to methacholine (MCh; acetyl β-methacholine chloride, Sigma-Aldrich, MO, USA) was assessed as previously described [38]. Briefly, we used a modification of the low frequency forced oscillation technique (FOT) to measure respiratory system impedance (Z_rs_) [31]. We fit the single compartment and constant phase models to Z_rs_ to generate respiratory system resistance (R_rs_) and to partition the impedance into airway resistance (R_aw_), tissue damping (G) and tissue elastance (H). Tissue hysteresivity (η) was calculated as the ratio of G/H [39]. Sensitivity to MCh was calculated as the dose required for respiratory system resistance, airway resistance, tissue damping and tissue elastance to increase by 150% (R_rs_EC_150_, R_aw_EC_150_, GEC_150_ and HEC_150_).

### Measurement of cellular inflammation and cytokines

At the conclusion of *in vivo* studies, bronchoalveloar lavage (BAL) fluid was collected by slowly washing 0.5 mL of saline in and out of the lungs three times via the tracheal cannula. The samples were centrifuged at 2000 rpm for 4 minutes and the supernatant collected for cytokine analysis by ELISA. The pellet was resuspended in phosphate buffered saline (PBS) and a 10 μL sample taken, stained with trypan blue and cells were counted using a haemocytometer. The resuspended BAL fluid was cytospun onto microscope slides and stained with Leishmann's stain for differential cell counts by light microscopy. Levels of BAL IL-5, IL-13, MIP-2 and IFN-α were measured using ELISA as per the manufacturer's instructions (BD Biosciences, San Diego, CA, USA). Total protein content was measured using a colorimetric assay (Bio-Rad, Gladesville, NSW, Australia) [40].

### Serum antibodies

Following *in vivo* measurements, blood was collected via cardiac puncture. Samples were centrifuged at 2000 rpm for 15 minutes and the serum taken for analysis of total IgE and HDM specific IgG_1_ levels by ELISA. HDM specific IgG_1_ was measured as previously described [27]. Levels of total IgE were measured as per the manufacturer's instructions (BD Biosciences, San Diego, CA, USA).

### Statistics

All data were analysed using SigmaPlot 12.5 (SPSS Science, Chicago, IL, USA). Lung function, inflammation and responsiveness to MCh were compared using two-way ANOVA with Holm-Sidak post-hoc tests. Data were transformed where appropriate and are reported as mean ± SD. p<0.05 was considered significant.

## Results

### Mass

Prior to experimentation, there were no differences in the mass of mice randomly allocated to the different treatment groups. HRV-1B infection resulted in significant weight loss (p = 0.013) in that infected mice were significantly lighter on the day of study (17.39±0.95 g) compared to the same mice on the day of infection (18.20±1.00 g) regardless of HDM-exposure. On the day of study, there were no differences in average mass between the different treatment groups (p>0.575 in all cases).

### Confirmation of HRV infection

**Figure 1:**
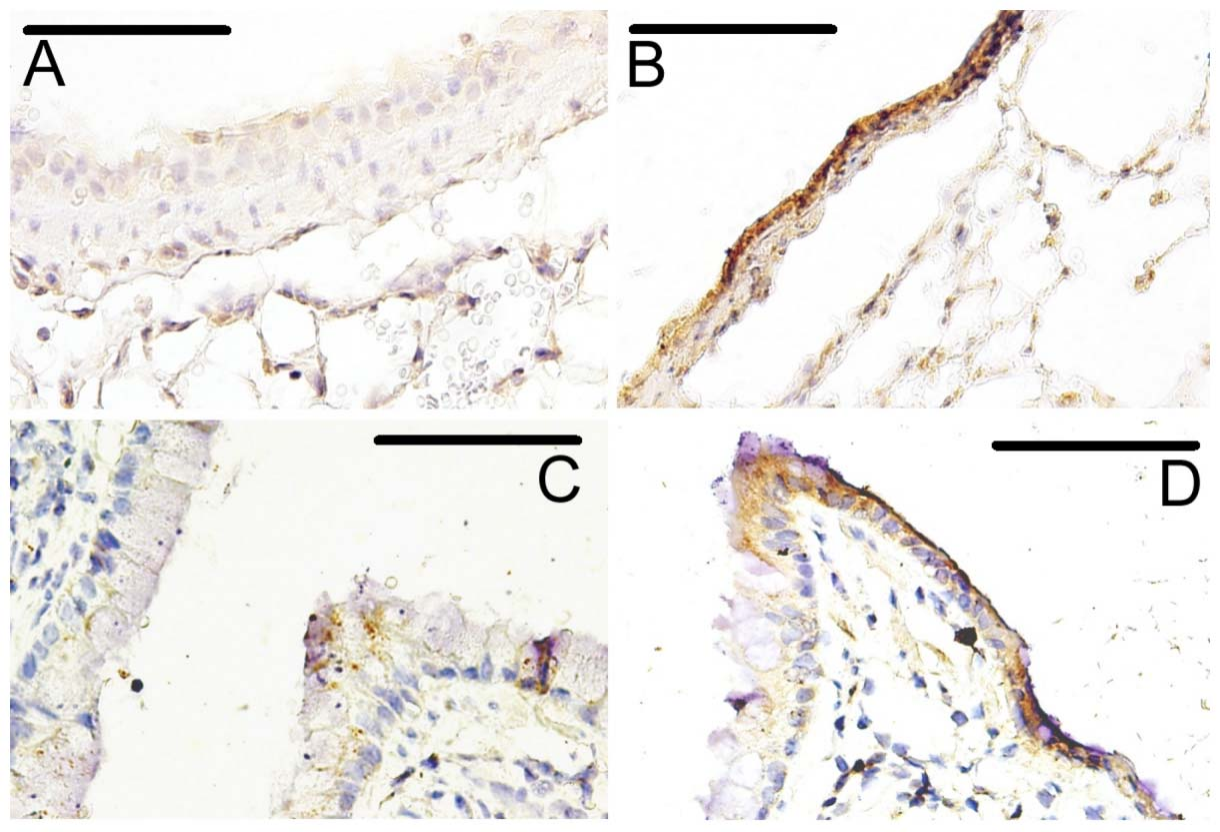
Variable lung pathology in mice infected with HRV-1B (or control) and exposed to HDM (or control). Adult female BALB/c mice were exposed to 25 μg of HDM protein (or control) daily for 10 days. On day 9 they were infected with 1×10^8^ TCID_50_ HRV-1B or inactivated virus. Samples were harvested 48 hours after infection. Representative images of immunohistochemical stained formalin fixed sections are shown - (A) Saline-iHRV, (B) Saline-HRV, (C) HDM-iHRV and (D) HDM-HRV. Infected mice show clear positive staining and an altered epithelium. Bars represent 50 μm.

Mouse lungs were formalin fixed, embedded in paraffin and stained for HRV-1B. Figure 1 shows typical staining patterns for uninfected (Figure 1A and 1C) and infected (Figure 1B and 1D) mice. Immunohistochemical staining specific for HRV-1B infection was identifiable in mice infected with HRV-1B (Figure 1B and 1D), but not in mice treated with inactivated HRV-1B (Figure 1A and 1C). HRV-1B infection had obvious cytopathological effects including epithelial cell shedding and possible dysruption of epithelial barrier integrity. As shown previously, HRV-1B staining was patchy and positive staining (brown colour) was observed in groups of epithelial cells in close proximity [21], [41]. There was no apparent visual difference in immunohistochemical stained lung sections obtained from Sal-HRV (Figure 1B) and HDM-HRV (Figure 1D) mice.

### Cellular inflammation

**Figure 2:**
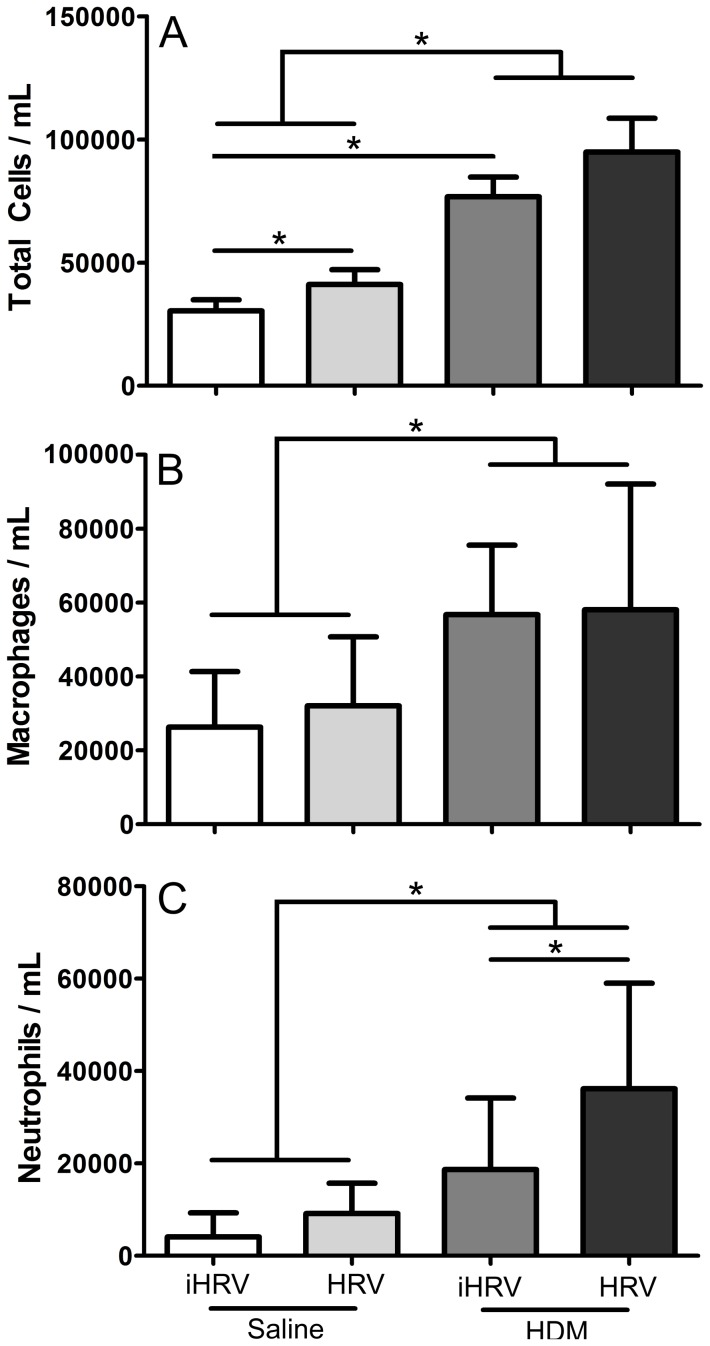
Exacerbation of bronchoalveolar neutrophilia by the combination of HDM exposure and HRV-1B infection. Adult female BALB/c mice were exposed to 25 μg of HDM protein (or control) daily for 10 days. On day 9 they were infected with 1×10^8^ TCID_50_ HRV-1B or inactivated virus. Samples were harvested 48 hours after infection. HDM exposure induced an increase in bronchoalveolar lavage total cells (A), macrophages (B) and neutrophils (C). HRV-1B infection alone increased total cellular inflammation (A). There was an additive effect of HDM exposure and HRV-1B infection on bronchoalveolar neutrophilia (C) * indicates a significant difference between groups. *n* = 8 to 10 per treatment. Data are mean ± standard deviation.

There was a significant interaction between HRV-infection and HDM-exposure with respect to total bronchoalveolar lavage inflammation (p = 0.009; Figure 2A). Mice infected with HRV-1B, but not exposed to HDM had greater numbers of total cells in their BAL compared with Sal-iHRV mice (41159±17814 cf. 30395±14280 cells.mL^−1^; p = 0.002), however there was no additive effect of HDM-HRV compared with HDM-iHRV (p = 0.639). The majority of cellular inflammation was macrophages, followed by neutrophils (Figure 2A and B). No eosinophils were detected.

There was a significant effect of HDM exposure on both macrophage (p<0.001) and neutrophil (p<0.001) numbers (Figure 2A and B), although there was no effect of HRV-1B infection alone on macrophages (p = 0.633) or neutrophils (p = 0.449). There was an additive effect of HRV-1B-infection and HDM-exposure on neutrophil numbers with HDM-HRV mice having significantly more BAL neutrophils (36149±22871 cells.mL^−1^) compared with mice exposed to either insult alone (Sal-HRV = 9125±6577 cells.mL^−1^ and HDM-iHRV = 18669±15477 cells.mL^−1^; p<0.012 in both cases).

### Serum antibodies

**Figure 3:**
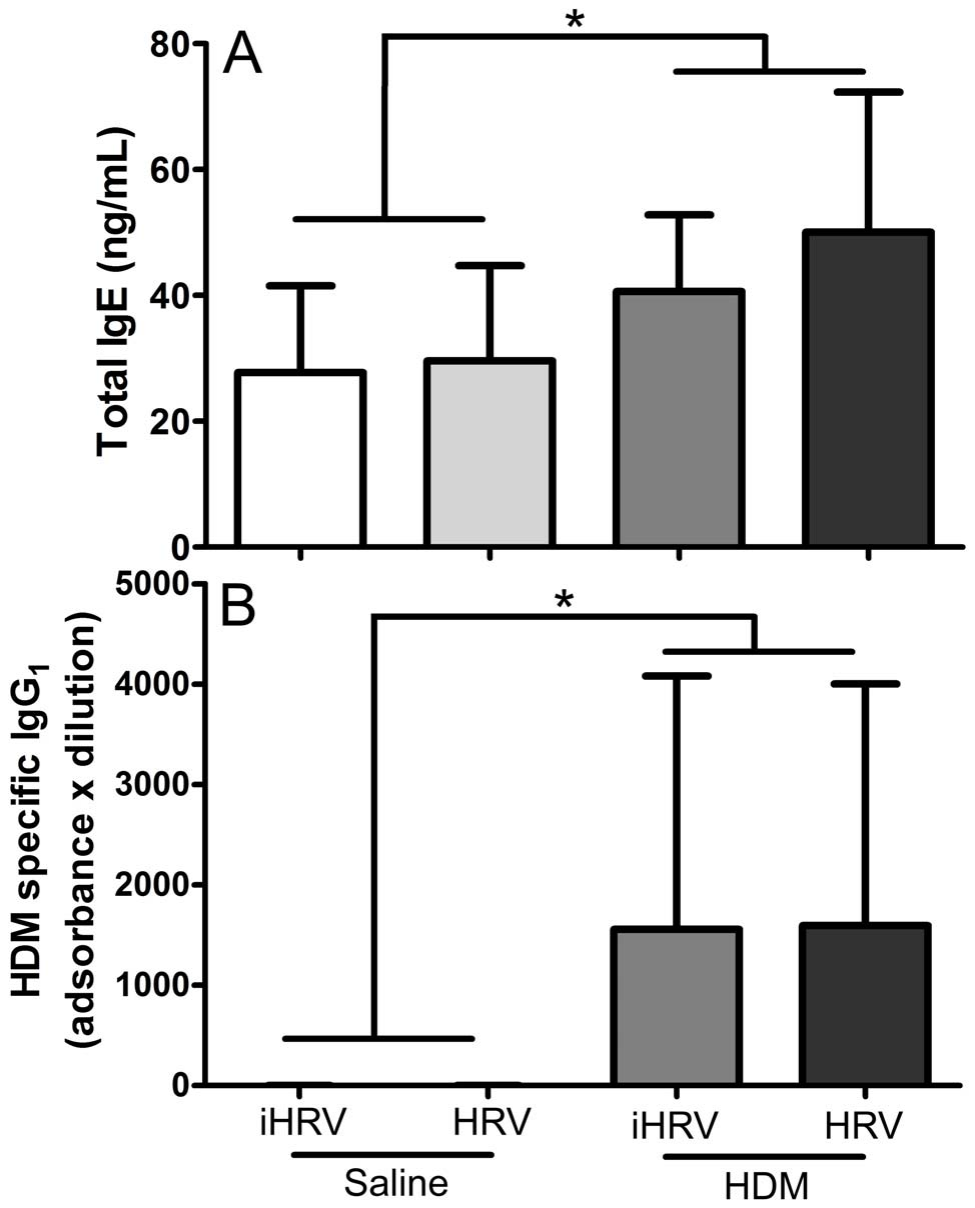
HDM exposure results in increased total IgE and HDM-specific IgG_1_. Adult female BALB/c mice were exposed to 25 μg of HDM protein (or control) daily for 10 days. On day 9 they were infected with 1×10^8^ TCID_50_ HRV-1B or inactivated virus. Samples were harvested 48 hours after infection. HDM exposure resulted in increased total IgE (A) and HDM-specific IgG_1_ (B), however there were no effects of HRV-1B infection, or the combination of HDM exposure and HRV-1B infection on serum antibodies. * indicates a significant difference between groups. *n* = 8 to 10 per treatment. Data are mean ± standard deviation.

Mice exposed to HDM had significantly higher total IgE (p = 0.041; Figure 3A) and HDM specific IgG_1_ (p = 0.009; Figure 3B) in their serum compared with non-exposed mice (Figure 3). There was no effect of HRV-1B-infection on levels of either antibody (p>0.250 in both cases), nor was there any interaction (p>0.675 in both cases).

### Mediators and protein

**Figure 4:**
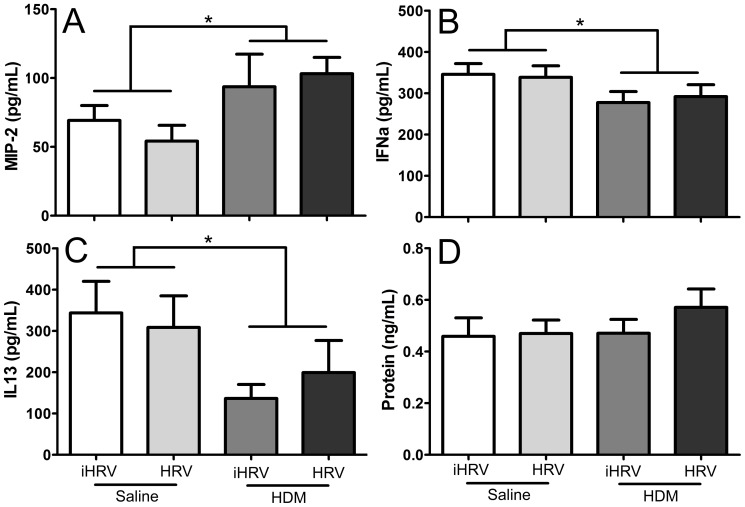
HDM exposure results in increased MIP-2 and decreased IFNα and IL-13. Adult female BALB/c mice were exposed to 25 μg of HDM protein (or control) daily for 10 days. On day 9 they were infected with 1×10^8^ TCID_50_ HRV-1B or inactivated virus. Samples were harvested 48 hours after infection. HDM exposure increased bronchoalveolar lavage MIP-2 (A), and decreased IFNα (B) and IL13 (C). There was no effect of HDM exposure on BAL protein (D), nor were there any effects of HRV-1B infection alone, or combined effects of HDM exposure and HRV-1B infection on any of these parameters. * indicates a significant difference between groups. *n* = 8 to 10 per treatment. Data are mean ± standard deviation.

There was a significant effect of HDM-exposure on levels of MIP-2 (p = 0.021; Figure 4A), IFNα (p = 0.044; Figure 4B) and IL-13 (p = 0.027; Figure 4C) in BAL (Figure 4). HDM-exposure was associated with lower levels of IFNα, IL-13, and higher levels of MIP-2 compared with saline-exposed mice. HDM exposure did not significantly alter total protein levels (p>0.105; Figure 4D). There was no effect of HRV-1B infection on IL-13, MIP-2, IFNα or protein levels (p>0.312 in all cases), nor was there any interaction between HRV-1B-infection and HDM-expsosure (p>0.418 in all cases). IL-5 was not detected in the BAL of any treatment group (limit of detection 55.25 pg/mL), consitent with the absence of eosinophils.

### Baseline lung mechanics

There were complex interactions between HDM-exposure and HRV-1B-infection with respect to baseline airway resistance (R_aw_) and tissue damping (G) (Table 1). Both HRV-1B-infection and HDM-exposure were required to cause a significant increase in baseline R_aw_. HDM-HRV mice had a significantly higher baseline airway resistance compared with HDM-iHRV and Sal-HRV mice (p<0.028 in both cases). There was no effect of either HDM alone (p = 0.874), or HRV1B-infection alone (p = 0.262) on baseline R_aw_. A similar effect was seen for tissue elastance (H), whereby HDM-HRV mice had significantly higher baseline H compared with mice exposed to either insult alone (p = 0.002). There was no effect of HDM alone (p = 0.598) or HRV-1B-infection alone (p = 0.528) on baseline G. HDM-exposure significantly increased baseline G (p = 0.009) and baseline respiratory system resistance (R_rs_) (p = 0.003), however there was no effect of HRV-1B-infection on either of these parameters (p>0.343 in both cases). Further, there was no interaction between HRV-1B and HDM with respect to baseline G. There was no effect of HRV-1B-infection or HDM-exposure on baseline hysteresivity (η), nor was there any interaction between HRV-1B-infection and HDM-expsosure for this parameter (p>0.07 in all cases).

**Table 1:** Baseline lung function as measured by the forced oscillation technique of mice exposed to HDM, infected with HRV or both.

	SaliHRV (n = 8)	SalHRV (n = 9)	HDMiHRV (n = 10)	HDMHRV (n = 10)
R_aw_	0.309±0.03	0.293±0.04	0.307±0.02	0.337±0.02^b^
R_rs_	0.516±0.06	0.510±0.07	0.558±0.05^a^	0.599±0.06^a^
G	6.17±0.88	5.99±0.96	6.49±0.65^a^	7.21±0.95^a^
H	36.20±7.03	34.14±7.05	38.24±5.27	44.95±8.45^b^
η	0.17±0.01	0.18±0.01	0.17±0.01	0.16±0.01

a  =  effect of HDM, b  =  combined effect of HRV and HDM. Data are mean ± standard deviation.

### Responsiveness to methacholine

**Figure 5:**
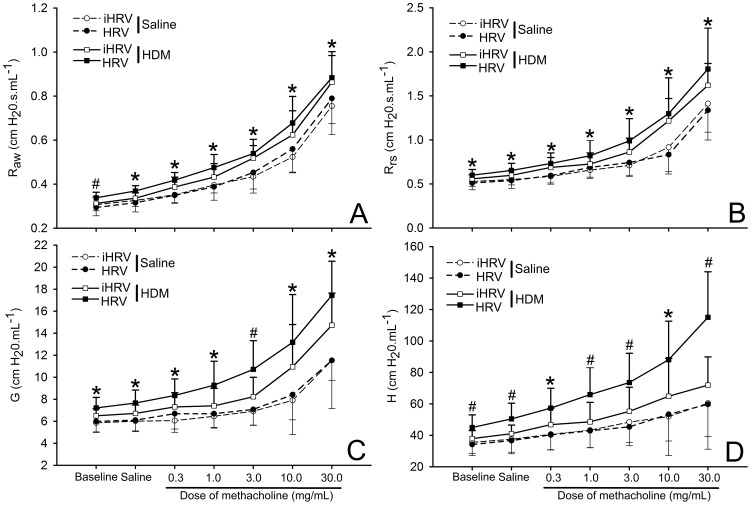
Exacerbation of parenchymal responsiveness to MCh by the combination of HDM exposure and HRV-1B infection. Adult female BALB/c mice were exposed to 25 μg of HDM protein (or control) daily for 10 days. On day 9 they were infected with 1×10^8^ TCID_50_ HRV-1B or inactivated virus. Measurements were made 48 hours after infection. We partitioned respiratory system impendence into airway resistance (A), respiratory system resistance (B), tissue damping (C) and tissue elastance (D). HDM exposure induced a greater response to MCh challenge with respect to airway resistance and respiratory system resistance. There was exacerbation of tissue responsiveness due to the combination of the two insults.* indicates a significant effect of HDM-exposure; # indicates an additive effect of HDM and HRV-1B. *n* = 8 to 10 per treatment. Data are mean ± standard deviation.

Responsiveness to MCh was significantly influenced by HDM-exposure, with HDM-exposed mice having significantly higher R_aw_ (Figure 5A), R_rs_ (Figure 5B) and G (Figure 5C) at the maximum dose of MCh (p<0.026 in all cases). There was no effect of HRV-1B-infection, or any interaction between the two insults for maximum R_rs_, maximum R_aw_ or maximum G (p>0.200 in all cases). There was, however, a significant interaction between HDM-exposure and HRV-1B-infection with respect to H at the maximum dose of MCh (Figure 5D) whereby HDM-HRV treated mice had a significantly higher H compared with mice exposed to either insult alone (p<0.001). There was no effect of either insult alone on maximum H (p>0.367 in both cases).

### Sensitivity to methacholine

**Figure 6:**
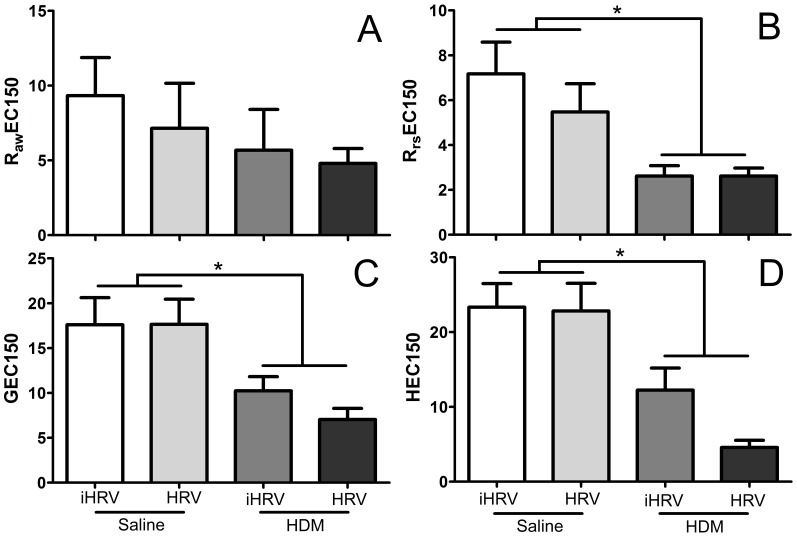
HDM exposure increases sensitivity to methacholine. Adult female BALB/c mice were exposed to 25 μg of HDM protein (or control) daily for 10 days. On day 9 they were infected with 1×10^8^ TCID_50_ HRV-1B or inactivated virus. Measurements were made 48 hours after infection. The dose of methacholine required to elicit a 150% increase in respiratory system resistance (B), tissue damping (C) and tissue elastance (H) was significantly lower in mice exposed to HDM compared to those not exposed to HDM. There was no effect of HRV-1B, or the combination of the two insults on sensitivity to MCh. * indicates a significant effect of HDM-exposure. *n* = 8 to 10 per treatment. Data are mean ± standard deviation.

Mice exposed to HDM had significantly lower R_rs_EC150 (Figure 6B), GEC150 (Figure 6C) and HEC150 (Figure 6D) values compared to non-exposed mice (p<0.001 in all cases). There was no effect of HDM-exposure on R_aw_EC150 (p = 0.176; Figure 6A), nor was there an effect of HRV-1B-infection on sensitivity to MCh for any parameter (p>0.159 in all cases). Although there was a trend towards the greatest sensitivity to MCh for HDM-HRV mice for all parameters, this was not significant.

## Discussion

The results of this study illustrate that HRV-1B infection can influence the response of adult mice to short-term HDM exposure. We did not see HRV-1B-induced exacerbation of “traditional” TH_2_ dominated allergic airways disease parameters such as airway responsiveness to methacholine, or bronchoalvolar lavage eosinophilia, but rather the additive effects of HRV-1B infection and HDM-exposure were in terms of increased neutrophilia and impaired parenchymal mechanics. These findings suggest that the model described in the current study could be more representative of a noneosinophilic asthma phenotype. This is supported by a number of murine studies of allergic asthma which propose a dissociation between airway hyperresponsiveness and “traditional” TH_2_ measures such as IL-5, IgE and eosinophilia [42], [43], [44]. Further, a recent study in humans shows that approximately half of patients with mild-to-moderate asthma have persistently noneosinophilic disease [45].

Combining short-term HDM-exposure and HRV-1B infection resulted in additive effects with respect to pulmonary neutrophilia, and some measures of lung function (Figures 2 and 5). While there was also a trend towards increased sensitivity to MCh for R_aw_, R_rs_, G and H, these were not significant (Figure 6). The additive effect of the two insults on BAL neutrophilia is commonly seen in similar studies employing ovalbumin [15], [16], [17]. Further, neutrophils are found in greater numbers in the nasal secretions, sputum, and BAL of allergic humans experimentally infected with un-purified HRV-16, compared with healthy controls [46], [47]. In humans, this neutrophilia correlates with increased IL-8 and airway responsiveness [48]. In our study, neutrophilia was associated with higher levels of MIP-2 (the mouse analogue of IL-8). These data support the notion that HRV infection may contribute to asthma exacerbation via augmentation of existing inflammation by enhancing the production of neutrophils and related chemoattractants [49]. Thus, while the pathology induced by the ovalbumin and HDM models is different, the apparent impact of HRV on disease exacerbation is similar. This suggests that viral exacerbation of asthma is not necessarily through traditional TH_2_ pathways.

We did not measure any further increase in airway hyper-responsiveness (R_aw_ as defined by the constant phase model) or respiratory system resistance (R_rs_ as defined by the single compartment model) in HDM-exposed mice also infected with HRV-1B, above that seen in mice exposed to HDM only. Previous studies combining HRV-1B infection with ovalbumin models of allergic airways disease [15], [16] have shown additive effects of systemic sensitisation and challenge with ovalbumin and HRV-1B infection on R_rs_. Unfortunately, these studies do not report changes in lung compliance or elastance and none use the constant phase model to partition respiratory system impedance into parameters representing the airways (R_aw_) and lung parenchyma (G and H). Instead, these studies use the widely discredited “enhanced pause” technique [50] or the single-compartment model to obtain an estimate of R_rs_. Respiratory system resistance includes a significant contribution from parts of the lung other than the main conducting airways, such that it is difficult to interpret from an asthma exacerbation (ie bronchoconstriction) point of view, if one assumes asthma is primarily an airways disease. Conversely, as R_aw_ is a measure of airway resistance, it would be expected to change if the airways are significantly narrowed [51], suggesting that, in our study, combining HRV-infection and HDM-exposure did not narrow the airways more than HDM-exposure alone. Instead, the combination of HRV-infection and HDM-exposure significantly increased tissue elastance (H). Changes in H have been interpreted as lung de-recruitment [52] and/or changes in the mechanical properties of the parenchyma [53]. In our study, the acute nature of the insults is suggestive of the former. This presents a potential physical mechanism for viral induced exacerbation of asthma symptoms and suggests that future research in this area, in particular those involving animal models, should consider more than just respiratory system resistance.

Despite infecting mice with a relatively high dose of purified HRV-1B (1×10^8^ TCID_50_), we saw very little effect of HRV-1B infection alone. In fact, the only parameter where HRV-1B alone had a significant effect was on BAL cellular inflammation (Figure 2). This was unexpected as experimental rhinovirus inoculation of human patients with asthma (or of healthy controls) has been shown to increase sensitivity to methacholine [54], [55]. The lack of a significant effect of HRV-1B infection on responsiveness or sensitivity to methacholine in our study limits the conclusions that can be inferred from our chosen model of HRV-1B infection. Bartlett et al (2008) showed that infecting adult BALB/c mice with a much lower dose of HRV-1B (5×10^6^ TCID_50_) resulted in significant changes in a range of lung inflammation and functional parameters [15]. However, the effects seen by Bartlett et al (2008) were often transient, usually peaking 24 hours after infection and resolving within 48–72 hours. We performed our measurements 48 hours after infection, based on preliminary kinetics studies (Figure S1) showing that this timepoint coincides with the peak of pulmonary inflammation in BALB/c mice. However, in taking measurements 48 hours after infection, we may have missed the relatively brief window of significant effects. Other researchers have infected adult mice with the same dose of HRV-1B used in the current study [16], [17]. Nagarkar et al (2009) showed that infected mice had significantly higher BAL neutrophils and were significantly more responsive (R_rs_) to inhaled methacholine up to 96 hours post infection [17] when this higher dose was used. This was not seen in a follow-up study, whereby HRV-1B induced neutrophilia had resolved 48 hours post infection [16]. As with our work, each of these previous studies used purified HRV-1B. The reasons for the relatively small and transient effects of HRV-1B infection in mice despite the high doses used are unknown. There appear to be unknown factors which limit viral replication in mice and result in a steady reduction in titre after infection [49]. This is despite the increase in vRNA 24 hours after infection, indicating positive viral replication. These findings, in conjunction with a lack of an increase in sensitivity to methacholine, strongly supports the notion that timing is critical in experiments involving HRV infection in mice.

Compared with mice infected with HRV-1B alone, much greater effects were seen in mice exposed to HDM alone, with HDM-treated mice exhibiting a range of physiological and immunological characteristics associated with allergen sensitisation. These included significantly increased BAL macrophages, neutrophils and MIP-2 in addition to increased serum total IgE and HDM-specific IgG_1_ (Figures 2, 3 and 4). Importantly, HDM exposure resulted in impaired baseline lung function and increased responsiveness/sensitivity to methacholine (Figures 5 and 6) 24 hours after the final exposure to HDM. The increased responsiveness to MCh was identified in R_aw_, R_rs_, G and H and hence was not restricted to the main conducting airways. In fact, HDM-exposure impaired tissue damping and tissue elastance more severely than it did airway parameters (Figure 5). This suggests that intranasal inoculation of mice with whole-crushed HDM in saline may not elicit a truly allergic response [56], but rather the pathology seen may be a consequence of repeated insult with a mixture of cysteine proteases capable of damaging the lung parenchyma [57]. Again, this implies that researchers should be careful to optimise their HDM model in relation to their specific outcomes of interest.

HDM exposure resulted in significant decreases in IL-13 (a mediator of allergic inflammation) and IFNα (released by leukocytes for antiviral immunity). Previous studies have demonstrated that asthmatic patients infected with HRV-16 (a major group serotype) had reduced type 1 interferons and IL-13 levels [12], [58]. This alteration in innate immunity could partially explain the increased frequency, severity and duration of lower respiratory tract symptoms in asthmatics when compared with healthy patients [59]. As previously mentioned, we did not observe the classical T-helper 2 type pathology induced by HDM exposure identified in previous studies [27], [60], [61]. For example, we did not detect any BAL IL-5 (a cytokine involved in the production, activation and survival of eosinophils) in HDM-exposed mice, and subsequently nor were any eosinophils identified in BAL. This result was unexpected as we replicated a model which has previously been shown to elicit a TH_2_ type pathology [60]. The HDM we used was 17.35% w/w HDM protein and contained 23.44 μg DerP/mg protein and 12.47 EU/mg of endotoxin. This is very similar to that employed by Gregory et al (2009) which contained 21.26 μg DerP/mg protein and 13.55 EU/mg of endotoxin. In the Gregory et al (2009) study, adult female BALB/c mice intranasally exposed to 25 μg of HDM protein in saline 5 days per week for 2 weeks had significantly increased airway resistance, lung and BAL eosinophilia and neutrophilia, IL-4, IL-5 and IL-13 compared to controls 24 hours after the final exposure [60]. The HDM composition, dose, timing and method of exposure employed by Gregory et al (2009) are all very similar to those used in the present study, such that discrepancies in results are difficult to interpret. Other studies have shown that HDM extracts with low serine and cysteine protease activity induce greater allergic sensitisation and asthma symptoms (including inflammation and increased TH_2_ cytokine levels *in vivo*) compared with extracts higher in proteases [62]. These data show that proteases in HDM extracts are not required *in vivo* for allergic sensitisation and eosinophilic inflammation and that the induction of allergic airways disease in mice is also independent of LPS levels. This is contrary to certain human data which show that the severity of asthma symptoms (including changes in FEV_1_ and the need for daily corticosteroid use) are directly correlated with the concentration of endotoxin in HDM. HDM extracts are complex mixtures, which induce both innate and adaptive immune responses and the properties of the particular HDM preparation used dictates the immune response and ensuing physiological responses [57]. The lack of eosinophilia and associated mediators may not be of critical importance in our model as the dissociation between pulmonary eosinophilia and AHR in allergic airways disease is well known, albeit controversial. Airways hyper-responsiveness has been shown to develop in BALB/c mice in the absence of airway eosinophilia [42], [43], and that high BAL eosinophilia does not necessarily correlate with increased AHR [44]. More recently it has been shown that eosinophils play a negligible role in the generation of HDM-induced allergic immunity and airway remodelling in mice [63].

In summary, the present study investigated the effects of HRV-1B infection on HDM-induced airways disease in adult female BALB/c mice. As seen in previous studies employing mouse models of HRV infection, the effects of HRV-1B infection alone were transient and considerably milder than the pathology induced by HDM alone. The mild pathology induced suggests that the mouse model of HRV-1B infection requires further optimisation, or that efforts should be focussed on developing a mouse model of HRV infection with a member of the HRV-C group. Further, while HDM exposure did not result in a classical TH_2_ driven response (such as BAL eosinophilia), there were significant effects of HDM exposure on most parameters measured, including increased cellular inflammation (primarily macrophages and neutrophils), AHR, increased lung tissue responsiveness, and increased total IgE and HDM-specific IgG_1_. This suggests that certain HDM-based models of allergic airway disease may be appropriate for recapitulation of the human noneosinphilic asthma phenotype. This is supported by our finding that the greatest additive effects of HDM and HRV-1B were with respect to increased neutrophilia. We also identified additive effects on lung parenchymal responsiveness and noted differences in airway epithelial structure between infected and uninfected mice. This supports the notion of HRV-induced damage to the airway epithelium may facilitate allergen sensitization and/or exacerbation of an asthmatic phenotype. In conclusion, acute rhinovirus infection was found to exacerbate house-dust-mite induced lung disease in adult BALB/c mice.

## Supporting Information

Figure S1 **HRV-1B infection induces a peak inflammatory response 48 hours after infection in mice.** Adult female BALB/c mice were infected with 5×10^6^ TCID_50_ HRV-1B in 50 μL DMEM, or UV-inactivated HRV-1B. Bronchoalveolar lavage fluid was obtained from separate groups of 8–10 mice on days 1–9, 14 and 21 after infection. Numbers of macrophages (A) and neutrophils (B) were determined by light microscopy as described above. * indicates a significantly greater number of cells compared to control. Data are mean ± standard deviation.
(TIF)
