# Supplementary figures and images for: Rhinovirus Exacerbates House-Dust-Mite Induced Lung Disease in Adult Mice
Source: PLoS One. 2014 Mar 14;9(3):e92163. doi: 10.1371/journal.pone.0092163 (PMC3954893; doi:10.1371/journal.pone.0092163)

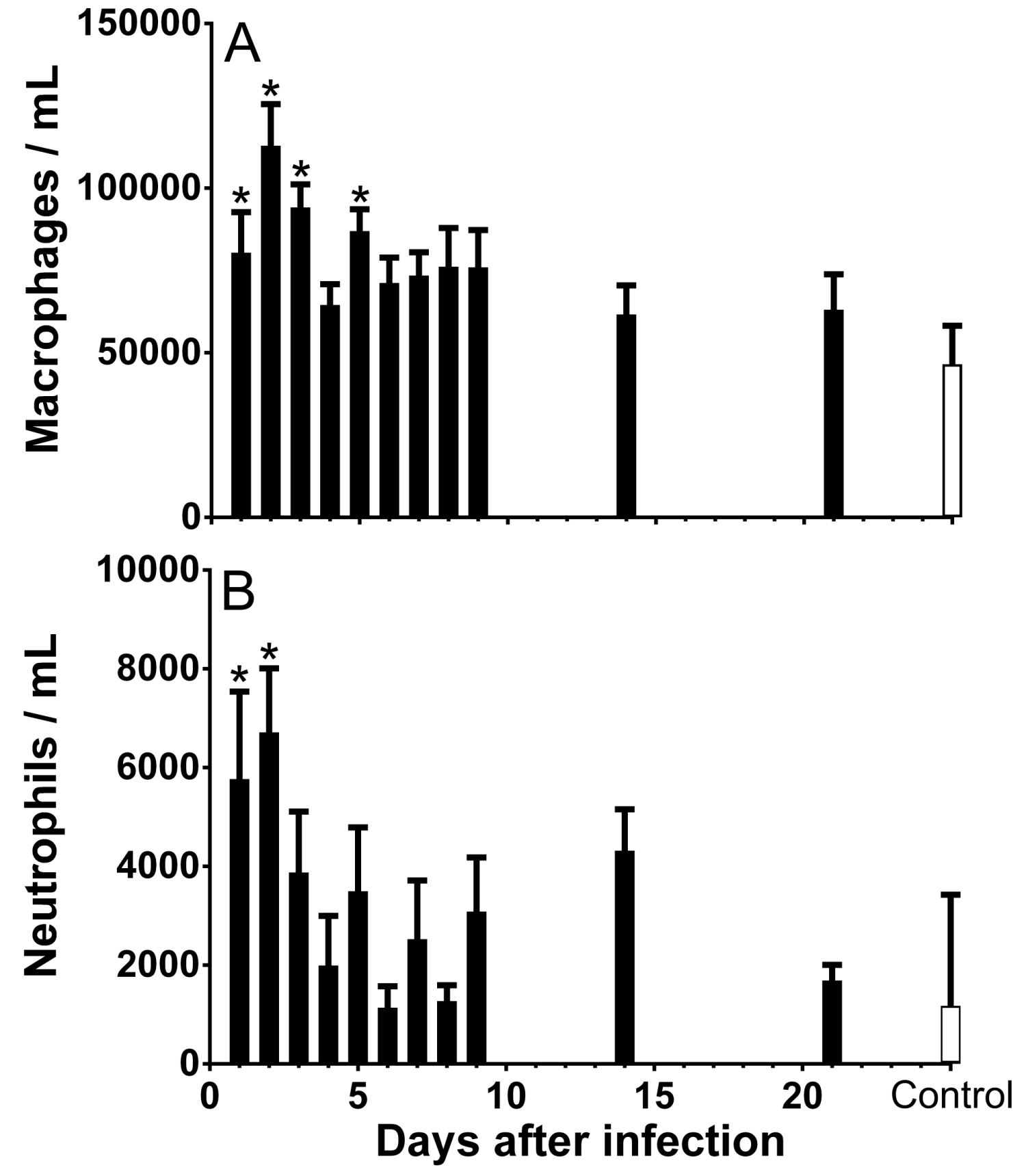

Supplement: Figure S1 — HRV-1B infection induces a peak inflammatory response 48 hours after infection in mice. Adult female BALB/c mice were infected with 5×10^6^ TCID50 HRV-1B in 50 μL DMEM, or UV-inactivated HRV-1B. Bronchoalveolar lavage fluid was obtained from separate groups of 8–10 mice on days 1–9, 14 and 21 after infection. Numbers of macrophages (A) and neutrophils (B) were determined by light microscopy as described above. * indicates a significantly greater number of cells compared to control. Data are mean ± standard deviation. (TIF) [file pone.0092163.s001.tif]
